# Retrospect of the Medical Sciences

**Published:** 1843-03-18

**Authors:** 


### FALSE JALAP.

At a meeting of the Philadelphia College of Pharmacy, held January 2, 1843, Mr. AUGUSTINE DUHAMEL submitted the Report of the Committee appointed to investigate a certain article of false jalap, brought from New York.

"Upon examination, it was found to be made up of the following pieces:

1st. A large spindle-shaped dried root, or rather tuber, flattened on one side, about six inches long and three wide, weighing six ounces.

2d. The larger half of a similar tuber, transversely cut, forming a segment four inches in its largest diameter, weighing three and a half ounces.

3d and 4th. Two entire tubers, smaller in size, ovate, one of them kidney-form and pointed, weighing together about five ounces.

A further description of this article is comprised under the following general features:

It is light in weight compared with jalap; externally very rugose, not minutely so, like the jalap, but coarsely furrowed: it is of a light brown color, with dark shades of black occupying the cavities, through which are interspersed minute shining black specks. Its fracture is rough and uneven, and its interior surface presents a uniform, grayish-white, ligneous appearance, and somewhat loose texture, marked by ash-colored concentric circles, composed of a harder and more compact substance indicating resin. One of the smaller tubers, wanting this distinguishing character, appears purely farinaceous. The taste and smell of these different tubers are feeble, sweetish, peculiar, and closely associated, though very distinct from jalap.

The largest root, divided transversely with a saw, exhibits vertical cavities, proceeding from incisions made through the whole length of the exterior surface to facilitate drying. In No. 2 the incisions are perceptible, but it has no holes like the other. Although a slight disparity exists in the internal appearance of these several tubers, yet their identity, in point of taste and smell, conclusively proves them to be of a common origin. The powder is grayish-white, and does not excite coughing or sneezing during pulverization.

This *drug* was represented to your Committee as coming from Mexico. A considerable quantity of it is to be found in the house of a drug broker in New York, who offers it for sale as *overgrown jalap root*, at a price little inferior to that which the genuine commands.

Your Committee are at a loss to determine from what plant it derives its source, as it bears no very close resemblance to the various adulterations to which jalap, as found in commerce, is known to be subject. It bears no analogy with the different specimens contained in the cabinets of our Professors of Materia Medica. Diligent inquiry among our druggists, (to whom it appeared novel,) led to no more satisfactory acquaintance with it, from which no doubt is left upon the minds of your Committee that the present is its first introduction into an American market.

It is evidently the produce of a *Convolvulus*, but of what particular species it is dfficult to say. It does not respond to the description of the dried root of the *C. panduratus*, nor any of the known falsifications furnished by this genus. The same observation applies to two varieties of adulterations mentioned by Guibourt in his *Histoire des Drogues*.

Contrasted with the well known characters of officinal jalap, (*Ipomœa jalapa*,) it presents the following discrepancies:

It is larger, lighter in comparison, wants brittleness, shining fracture and compactness, acridity of taste, odor, and color. It is also deficient in resin, and wants the striated and reticulated appearance of exterior which the other possesses.

In order to ascertain how its chemical relations would comport with jalap, a number of experiments were conducted by the Chairman of your Committee, the result of which enables them to furnish a proximate analysis of its composition as follows. In juxtaposition are placed the analyses of officinal and male jalaps.

*False Jalap.*

| | |
|---|---|
| Resin, consisting of 15 soft and 20 of dry brittle resin, | 35. |
| Gummous Extract, | 85. |
| Starch mixed with Inulin, | 140. |
| Lignin, | 116. |
| Albumen and Gum, | 50. |
| Saccharine Matter, Salts of Lime, and loss, | 74. |
| | 500 |

*Analyses from* 500 *parts.*

| *Of Jalap, by Cadet.* | | *Of Male Jalap, by Ledanois.* | |
|---|---|---|---|
| Resin, - - - | 50 | Resin, - - - | 40 |
| Gummy Extract, - | 220 | Gummy Extract, - | 128 |
| Fecula, - - - | 12 | Fecula, - - - | 16 |
| Lignin, - - - | 145 | Lignin, - - - | 290 |
| Albumen, - - | 12 | Albumen, - - | 12 |

Having proceeded thus far, it remained for yonr Committee to ascertain its medicinal properties, if, after what is here related, it could be supposed to possess any. This they were enabled to do through the courteous offer of Prof. Dunglison. A trial of its virtues was made at the Blockley Hospital, under the inspection of some of the resident faculty, upon six different individuals, in doses of fifteen to twenty grains, without obtaining any effect whatever."—*American Journal of Pharmacy*, Jan., 1843.

---

*Taliacotian Operation.* By J. MASON WARREN, M. D. of Boston.

The patient was a lady, 30 years old. Having a little warty excrescence on the nose, she applied to a quack, who happened to enjoy considerable notoriety at the time in the treatment of cancers, and who, as usual, at once informed her that the disease was cancerous, and advised its removal. A caustic was applied, but so badly managed, that not only the dissease, but a portion of the nose also was destroyed, leaving the unfortunate subject in a most distressing situation.

It may, perhaps, be remembered, that in a former case, which was published in this Journal, an operation was required under precisely similar circumstances; and they may serve as a warning to those who are so led away as to submit themselves to the mercy of ignorant charlatans.

This lady, when I first saw her, some time after the ulceration from the effects of the caustic had healed, looked exactly as if the nose had been neatly excised; the skin, a portion of the cartilage forming the septum nasi, and about one-third of the columna were wanting. The nasal cavities were thus quite exposed, and the deformity produced of a very striking and disagreeable character.

I at once perceived that the Indian method would not be applicable, as it required too great a sacrifice in bringing the integument from the forehead, and I determined to adopt the Italian method of transplanting the skin from another part of the body. In a former case, already referred to, where the skin was taken from the fore-arm near the wrist, an obstacle presented itself, which had not been fully foreseen; this was the impossibility of retaining the arm sufficiently steady, by any apparatus that could be devised, to prevent the movements of the body being communicated to the part where the uniting process was going on; by the method of Taliacotius, the head and arm are kept immoveably confined during the time of union, and no motion of the patient can disturb these parts if the bandage remains firm. This position may easily be supported by a thin subject, but in a large muscular man it is next to impossible even to bring the arm into the proper position, much less to preserve it there for any length of time. The present case being considered a proper one for the latter method, it was determined to attempt it.

I advised my patient to return home, and to have a bandage made such as described and depicted in the work of Taliacotius, and to exercise herself daily for a few weeks in keeping the arm up in contact with the face in the position which it would be requisite to maintain after the operation. This was done, and all preparations being made, the operation was performed on the 21st of October, 1840, in the presence of Dr. Reynolds, Dr. Townsend, Dr. Inches, and a number of other medical gentlemen.

The cicatrix covering the edge of the nostrils was first removed, and the apex of the septum and columna nasi made into a raw surface. A flap, nearly double the size required, was now dissected out from over the upper part of the biceps muscle of the right arm, its base, which presented downwards, being left attached. The bleeding having ceased, and the flap contracted, which it did nearly one-half, the arm was brought up to the face, and the edges of the flap confined in contact with the raw surface of the nose by six sutures. The bandage of Taliacotius was now applied, and served to maintain the arm immoveably fixed in contact with the head. The whole of this painful operation was supported with the most determined fortitude.

October 22d.—Since yesterday she has remained in an arm-chair, preferring the sitting posture as the most comfortable, both for breathing and for taking nourishment. For an hour or two after the operation, the arm was quite numb from its constrained position and the pressure of the bandages; this gradually changed to a painful sensation. She now has some pain in the elbow, none in the shoulder, where it would have been most expected, and less than yesterday in the fore arm. Her pulse is good, she has slept at intervals, and takes gruel with appetite, sucking it through a glass milk tube, which was the best contrivance we could think of for this purpose; she has left her chair and walked about the room without disturbing the bandages.

23d.—She complains to day of severe pain in the wrist, which was very soon relieved by wetting the bandages with laudanum; and almost immediately after each application, when it was required during the day, she was composed to sleep. The bandages were relaxed a little from being wet, but not so much as to injure the adhesion.

24th.—At 10 o'clock, 72 hours after the operation, I proceeded, in presence of a number of medical gentlemen, to divide the pedicle, and release the arm from its painful position. On first letting it down, the arm appeared quite paralyzed, but by gentle friction the power of motion and sensation was gradually restored.

A perfect adhesion had taken place between the new flap and the right side of the nose. On the other side, the skin was so wrinkled up, from the pressure of the head downwards on the arm, that it was not possible to determine what was the state of union. Out of the new flap a pedicle was now shaped, to serve for the completion of the columna, and was confined in contact with what remained of the old one, by a single suture.

The patient was in good spirits, and appeared but little fatigued from the painful position in which she had been confined for such a length of time. Her sufferings had certainly been greatly alleviated by the possibility of being able to move about the room without interfering with the adhesive process, owing to the perfect retentive power of the bandages.

25th.—Quite comfortable; the tip of the nose looked well; the edges on one side somewhat livid, but on being touched with the knife bled freely; a portion of the new columna in a sloughing state.

November 11.—A small piece of the skin which formed the septum having sloughed, the remainder has settled down, and at present is firmly united in its situation. The nose has a good shape, but is still a little swollen.

December 12th.—This patient returned home well. Nose has entirely healed; its form is good; the tip is slightly turned up, and the whole organ a little shortened when compared with its original dimensions; but is still agreeable, and presents nothing remarkable to a casual observer; the line of union has so melted down into the surrounding parts as to be scarcely perceptible. She was seen by a number of physicians before leaving town, who were able to congratulate her on the successful termination of the operation.

At the present time more than two years have elapsed, and no unfavorable change has taken place in the aspect of the restored organ, and the patient has had no reason to regret the suffering she has undergone.—*Boston Medical and Surgical Journal*, March 1, 1843.

---

*Observations on a particular Form of Encysted Tumour, which occurs in the Neck, but is not necessarily connected with the Thyroid Body.* By Benjamin Phillips, F. R. S., Surgeon to the St. Marylebone Infirmary, &c.

Mr. Phillips describes what Maunoir of Geneva had described before as Hydrocele of the Neck, and has been described also by Mr. Hill, of Dumfries, Dr. O'Beirne, and others. The following is his summary drawn from the cases of all.

"The cases which I have detailed, as well as those described by the authors I have named, justify me in stating, that there is a class of unilocular or multilocular encysted tumors developed in the neck, which contain a serous fluid, varying from a light yellow to a deep coffee-color; that in its nature this fluid is albuminous, coagulable by heat; that those tumors are generally developed, quite independently of the thyroid body, though in their progress they may become intimately connected with it, but that the connection does not usually involve any change of structure in that organ; that they are most frequently developed at, or after, the middle period of life; that they occur almost indiscriminately in both sexes; that they are almost always of slow growth, and that they often attain a large size; that they do not usually prove troublesome, until they are of sufficient size to interfere with respiration or deglutition. That the treatment by simple puncture, so as to evacuate their contents, is usually insufficient to cure the disease, becaase the opening is apt to close up and the cavity to refill; that puncture, with injection, has not succeeded better, and for this reason, either the fluid is too stimulating, and produces violent inflammation, with spasmodic action of the organs of respiration and deglutition, or it does not exercise sufficient action to modify the surface of the sac; and it is not easy to ascertain the just medium; that the treatment by making an incision of sufficient extent to expose the greater part of the interior of the sac, and stuffing the cavity, has been followed by too much local and constitutional irritation to render it prudent to adopt it as an ordinary method of cure; that the plan which has succeeded best is that to which Mr. Hill, of Dumfries, resorted, and which has been employed by M. Maunoir, Dr. O'Beirne, and Petrale, namely, puncture and seton. Whether an ordinary tent passed through the long diameter of the sac, after puncture, might not answer the purpose equally well, we have not yet the necessary experience to determine, but I see no reason why it should not succeed."

He relates, himself, two cases. In the first, the cyst discharged itself through a puncture, made with an exploring needle. The fluid, in the first instance, was thin and bloody, then became bright red, and reduced the strength of the patient as well as the size of the tumor. Next the discharge became sero-purulent, and lastly purulent. It stopped under the influence of time, *or* quiet and swallowing ice. The patient recovered, the tumor having lessened very much, but the thyroid body remaining enlarged.

In the second case, the tumor cracked spontaneously, and about three pints of reddish serous fluid escaped. On the following night, three pints more escaped. A sero-purulent discharge flowed for many weeks, when she recovered, an inconsiderable enlargement of the thyroid body remaining. Twelve months afterwards there had been no re-accumulation of fluid in the sac, but the enlargement of the thyroid body still continued, and a small fistulous communication with the sac remained. We fancy that this affection is not very uncommon.—*Med. Chir. Review*, Jan., 1843.

---

*English Physicians.* By PROF. MARX.

The English physicians know little of scientific medicine. They are wanting in systems. With them theory has not interpenetrated experience. Anything of the kind they display is no more than an insipid eclecticism. They think little of arrangements and deductions founded on or flowing from fundamental principles. Their science is not the product of pure thought; it is the mosaic work of observations. If subjects of the kind be brought under the notice of an Englishman, he scarcely understands you; and when you try to make him comprehend your meaning, he will say, we must be just before we are generous. They insist that people should busy themselves with substantial realities, and not fine-sounding words. The construction of philosophical theories, and the dressing up and arrangement of facts and general propositions, are certainly not their forte. But it would be matter for regret if they were to fritter away their sound sense, enlightened judgment, and innate truthfulness in an infinity of subtle distinctions; or become mystified by the opinions of the theorizing metaphysical school. But in fact, their native love of freedom repels the stays and straight-waistcoats of a system. Inhabitants of a country which carries on an intercourse with the whole world, they are accustomed to a wide horizon, and from an early period are practised to distinguish the apparent from the real. They think that this is peculiarly the duty of the physician; that he must examine each fact in its varied and almost infinite relations as clearly, accurately, and precisely as possible; and then arrange the results logically, expressing them under plain and perspicuous propositions. Poets only may be permitted to weave the silken threads of imagination, and philosophers plunge into the depths of pure thought to bring up mere idealities, useless fantasies and systems, or wander after cold and desolate generalities. I had already formed opinions of this kind respecting the English physicians, and the tendency of their scientific pursuits, from a perusal of their writings. A personal acquaintance with them at the bed-side, in their public institutions, and in their schools, has only served to fix and extend these opinions.—*British and Foreign Medical Review*, January, 1843.

---

NERVOUS OPHTHALMIA.

The affection so named by M. Lisfranc is a good example of local *irritation* in contradistinction to an *inflammation*; in other words, perverted action of the nerves, rather than of the blood-vessels of the part. The symptoms are lachrymation; no morbid appearance in the interior of the eye or on the cornea; marked photophobia; the eyelids carefully shut; on opening the eyelids, the patient feels as if points of nails were being driven into the globe; the conjunctiva is slightly reddened, but does not appear swollen. In the treatment, antiphlogistics fail. Extract of belladonna is to be rubbed on the temples and around the base of the orbits night and morning; sometimes the same medicines may be administered internally. In a few days the cure is complete. Finding such irritations of a mucous surface externally, closely simulating the state of inflammation, M. Lisfranc is naturally led to conclude that probably similar conditions and simulations may not unfrequently exist internally; and that many pains in the stomach, bowels, urethra, &c., supposed be inflammatory, and yet resisting all antiphlogistic and revulsive means, may be dependent on mere irritation, and ready to yield almost at once to narcotics. We agree with M. Lisfranc in thinking this extremely probable.—*Ibid.*

---

*New mode of Extraction of Cataract.* By DR. BONNET, of Lyons.

He lays the patient on his back. If it is the left eye, he places himself at the patient's right side, and having applied the elevator and depressor to the eyelids, entrusts them to two assistants, who must see that they do not press upon the eyeball. With his left hand, the operator holds a common pair of forceps; with his right, a hooked spring pair. With the former, he raises the conjunctiva into a fold, and with the latter takes firm hold of that membrane, and of the subconjunctival fascia about one-sixth of an inch from the cornea, a little above the external angle of the eyelids. This forceps is then entrusted to the assistant who supports the upper eyelid; it fixes the eye aud prevents it from rolling inwards. The operator now takes the depressor of the lower lid into his left hand, and with the right uses the knife to divide the cornea.

To the mode of fixing the eye, above explained, he ascribes the promptitude, precision, and facility with which the section is accomplished. The incision is always parallel to the edge of the cornea; the iris is never wounded; the operation is as simple, he says, as bleeding: nò dangerous consequences ever arise from the pinching of the conjunctive with the forceps; the specula applied to the eyelids prevent pressure on the eyeball, and that dangerous expulsion of vitreous humour which is apt to happen when the fingers are employed to fix the eye and separate the eyelids! All this we owe to the operation for strabismus!

Dr. Pétrequin employs Bonnet's method of fixing the eye, in the operation of displacement, and in those for artificial pupil.—*Ibid, from Annales d'Oculistique.*

### *On the Chemical Composition of the Fluid of Ranula.* By L. Gmelin.

It is entirely different from saliva, containing no sulphocyanate, and only a small quantity of salivine, but chiefly albumen. The albumen, however, amounts to only 2 per cent., though the fluid is as thick as others containing about 5 per cent. It is to be supposed, therefore, either that the fluid of ranula contains some peculiar material to which it owes its thickness, or else that the albumen in it is of some peculiar kind.—*Ibid, from the Annalen der Chemie,* 1842.

### *On the Fractures of the Lower End of the Radius.* By M. Voillemier.

The chief object of this very excellent paper is to prove, especially by examination after death, the direction which fractures of this kind usually take, and thence to deduce the best method of treating them. The difficulty and importance of the question is sufficiently proved by the number of essays which have been recently published upon it. We can only abstract the peculiar portions of the author's views.

As the basis of these he points out some facts in the structure of the radius, namely, the double inclination of its inferior articular surface, which is oblique from above downwards and from before backwards, and from within outwards; its prominent border behind and on the outer side; and, above all, the thinness of the compact tissue of the lower extremity of the radius, when compared with that of the shaft, the former becoming suddenly almost as thin as paper, while the latter is so thick that there is scarcely any medullary tube. It is from this last circumstance that there results both the peculiar tendency to fracture near the wrist, and the position in which, according to M. Voillemier, the fragments are usually placed, and which is such that the fracture may be called *fracture by penetration.*

In a fall on the hand (the most frequent cause of this fracture,) all the force of the shock is transmitted to the radius, and it breaks at its weakest point, where the compact tissue is thinnest, and *that* is just above the wrist-joint. At this part, too, the cancellous tissue is first abundant; the compact tissue of the wall of the upper portion is, therefore, by the continuance of the force after the fracture has taken place, driven into the cancellous tissue of the lower. This penetration may take place in several ways. If the extremity of the bone is large, and the force has been transmitted in nearly the direction of its axis, and all parts of the wall have yielded almost simultaneously, the whole of the end of the superior fragment may be driven into the lower one, and the two pieces may be thus locked or *nailed* one within the other. When, in such a case, the force is very great, the upper fragment may be so driven into the lower, that the latter may be completely smashed; it was to cases like this that Dupuytren gave the name of *fractures par ecrasement.*

But this mode of fracture, as might be expected from the number of circumstances which must combine to produce it, is rare; the more common form is produced when the force of the fall comes somewhat obliquely upon the radius. In this case, the line of fracture is, as in the last, nearly parallel to the plane of the articular surface, but since the force falls more on the posterior than on the anterior part of the latter, it follows that the posterior edge of the fractured compact tissue of the upper fragment begins first to penetrate into the cancellous tissue of the lower, and that the anterior edge, instead of penetrating, slides over upon the dorsal surface of the lower or carpal fragment. There is thus a dovetailing or reciprocal penetration of the two portions. And a similar arrangement of parts is seen, whether one traces them from behind forwards, or from without inwards. In the former direction, one comes first on the dorsal portion of the upper fragment, then on the same portion of the lower fragment, and then, in the same succession, on their palmar portions; and in the latter direction there is, on the outside the outer margin of the lower portion, then the outer margin of the upper one, and so on; for the outer edge of the upper portion in these cases always penetrates the cancellous tissue of the lower in such a manner that, if the line of its wall were carried on, it would cut off the styloid process. Hence it is that in all the cases of fracture of the radius near the wrist which M. Voillemier has examined long after the accident, he has found on a vertical section of the bone, a line of compact tissue continued straight downwards from the outer wall of the shaft into the interior of the cancellous tissue of the head. There are not two such lines of compact tissue, because that of the lower portion which should penetrate into the upper, is usually broken up and absorbed, in consequence of its extreme thinness.

Having found this state of parts in the great majority of instances, M. Vollemier denies the existence of oblique fractures of the lower end of the radius, and of those especially which M. Diday had described as fractures "tailles en biseau." These, he says, have been supposed to exist in consequence of the sensation which fragments, placed as described above, give when examined during life. The dorsal edge of the upper fragment projects somewhat lower down than the palmar edge of the lower one, for the former is pushed downwards over the lower fragment, the latter upwards over the upper one; and hence it seems to the touch as if the line of fracture were oblique from the palmar to the dorsal surface, and from above downwards. But such a state of parts has never been found on dissection. The condition is analogous in those rarer cases in which the lower fragment passes, not towards the dorsal but towards the palmar aspect of the upper one. Here there is only a seemingly oblique direction of the fracture, in consequence of the projecting edge of one fragment being higher up than that of the other.

From these pathological facts, which we believe to be exactly conformed to the truth, M. Vollemier gives a clear *rationale* of the symptoms, and then deduces the mode of treatment best to be followed.

The chief feature of that which he recommends is its simplicity, but he, without doubt, asserts a true principle when he says that in these fractures, even more than in most others, the apparatus must be adapted differently to almost every case.

An important addition to this paper is that part in which M. Voillemier speaks of the separation of the inferior epiphysis of the radius which may take place in consequence of severely twisting the wrist-joint. He was led to notice this, by having accidentally produced it in an attempt to dislocate the wrist of a dead body; and he subsequently convinced himself that either this separation, or a fracture of the lower end of the radius, without complete rupture of the surrounding fibrous tissues, might be thus produced during life. He relates two cases in which he believes it had occurred, and points out, as its chief signs, the absence of deformity where all the other characters of fracture are present, and a most severe pain at the ordinary situation of the boundary between the epiphysis and shaft, whenever any attempt at motion is made.—*Ibid, from Archives Générales de Medecin.* Mars, 1842.

### PREVENTION OF LEAD COLIC.

Mr. Benson, the managing director at the British white lead works, Birmingham, says that the use of what he calls sulphuric beer by the workmen is an effectual preventive of the colic arising from the effects of the lead, and which, previously to the employment of the sulphuric beer, was exceedingly prevalent among the men. He was induced to try it from a statement made some time since, that sulphuric leamonade had been successfully used at a white lead manufactory in France. Its action must be effected by the chemical transformation of the poisonous carbonate into the innocuous sulphate of lead. The formula for the preparation of the beer is as follows;—Take of treacle, fifteen pounds; bruised ginger, half a pound; water, twelve gallons; yeast, one quart; bicarbonate of soda, one ounce and a half; sulphuric acid, one ounce and a half by weight. Boil the ginger in two gallons of water; add the treacle and the remainder of the water hot. When nearly cold, transfer it to a cask, and add the yeast, to cause fermentation. When this has nearly ceased, add sulphuric acid, previously diluted with eight times its quantity of water, and then the bicarbonate of soda, dissolved in a quart of water. Close up the cask, and in three or four days the beer will be fit for use. As acetous fermentation speedily takes place, particularly in hot weather, new supplies should be prepared as required. The object in adding the bicarbonate of soda is to give a pleasant briskness to the beverage. The sulphuric acid remains greatly in excess.—*Lancet*, Dec. 17, 1842.

### ROBERTSON ON THE CAUSE OF CARIES OF THE TEETH.

Prior to the appearance of the first edition of Mr. Robertson's treatise, in 1835, the prevailing opinions concerning caries of the teeth were those of Fox and Bell; the former maintaining that the disease consists in an inflammation of the bony substance of the crown of a tooth, which secondarily affects the lining membrane of the organ, causing its separation, and a consequent decomposition of the solid parts; the latter insisting that caries commences in inflammation of the bone immediately under the enamel, from which, he says, the tooth, owing to its imperfect vitalization, cannot recover, and death and decay are the consequence.

Mr. Robertson, on the other hand, maintains that the remote cause of destruction of the teeth is the decomposition of food which lodges in the interstices between them.

The inflammatory theory, then, is to the effect that the teeth decompose by vital action, commencing in their interior, and proceeding to their surface. Our author's theory is exactly the opposite, namely, that decay begins by chemical action upon the surfaces of the teeth, and proceeds to their interior, and that inflammation is the consequence, and not the cause, of caries. His arguments are chiefly derived from the fact, that the teeth decay only in such situations as are favourable for the lodgment and decomposition of food, and never upon their smooth and even surfaces; from their decaying in pairs, and at particular periods of life; from the ready relief which filling and filing afford, if decay be not too far advanced; from the disease commencing externally and proceeding inwards, and never conversely; and from the circumstance, that artificial teeth are liable to the same species of destruction as natural ones.

These arguments we apprehend to be perfectly sound, and in accordance with the rules of common sense and experience; for we cannot imagine a disease, which owes its origin and progress to inflammation, to be susceptible of alteration or cure by means which, of all others, are most calculated to contribute to such action. But when the cause is purely chemical, and owing primarily to a defect of structure, it is not irrational to suppose that mechanical treatment will arrest or remedy it. It is on this ground that we think the practical application of Mr. Robertson's theory particularly valuable. Believing, as he does, that inflammation is not the cause of caries, but the consequence of it, and dependent upon the influence of atmospheric and other agencies upon the lining membrane of a tooth, he urges the necessity of removing any decay that may be apparent, *before the occurrence of pain;* for after its commencement there is little hope of a permanent remedy. He insists upon the propriety of constantly using a tooth-brush, so that any particles of decomposing food may be removed; and of an occasional inspection of the teeth by a dentist, that any lurking decay may be arrested. He tells us that these suggestions have wrought a considerable change in his own practice—that people now apply to have caries arrested, rather than to have teeth removed in consequence of its ravages; and he maintains that, if this plan were universally acted upon, tooth-ache would be comparatively unknown, and the organs would be preserved in beauty and usefulness to the remotest period of life.—*London Med. Gaz.* Dec. 9, 1842.

### REVIVAL AFTER FREEZING.

In the winter of 1828-9 in Ireland, Gaimard found that toads could be completely frozen, so that ice lay in small pieces between their muscles; their bodies became quite hard, stiff and motionless, broke easily and without any effusion of blood, so that, in short, every trace of life disappeared, and yet in ten or twelve minutes they could be revived by immersing them in very slightly warmed water. If they were too quickly frozen they did not revive.—*Brit. and For. Med. Rev. from Bib. Universelle.*

